# The Peer Review Process: Past, Present, and Future

**DOI:** 10.3389/bjbs.2024.12054

**Published:** 2024-06-17

**Authors:** John A. Drozdz, Michael R. Ladomery

**Affiliations:** Department of Applied Sciences, University of the West of England, Bristol, United Kingdom

**Keywords:** peer review, single and double blind peer review, triple blind peer review, transparent peer review, open access publications

## Abstract

The peer review process is a fundamental aspect of modern scientific paper publishing, underpinning essential quality control. First conceptualised in the 1700s, it is an iterative process that aims to elevate scientific literature to the highest standards whilst preventing publication of scientifically unsound, potentially misleading, and even plagiarised information. It is widely accepted that the peer review of scientific papers is an irreplaceable and fundamental aspect of the research process. However, the rapid growth of research and technology has led to a huge increase in the number of publications. This has led to increased pressure on the peer review system. There are several established peer review methodologies, ranging from single and double blind to open and transparent review, but their implementation across journals and research fields varies greatly. Some journals are testing entirely novel approaches (such as collaborative reviews), whilst others are piloting changes to established methods. Given the unprecedented growth in publication numbers, and the ensuing burden on journals, editors, and reviewers, it is imperative to improve the quality and efficiency of the peer review process. Herein we evaluate the peer review process, from its historical origins to current practice and future directions.

## History of Peer Review and Contemporary Challenges

### Origins of the Peer Review Process

Accurate and effective scientific writing is an essential part of the modern scientific method. It is required for the optimal communication of research to peers and to a wider scientific community [1]. The history of scientific writing is complex; the roots of modern conventions in England trace to the 8th century, when Anglo-Saxon monks such as Bede are observed to discuss and disseminate medical writing published by themselves and by European contemporaries [2, 3]. Between the 14th and 15th centuries, scientific writing became much more widespread [2]. What is now understood as the “scientific journal” dates to the 17th century, with the founding of The Royal Society in 1,660 and the launch of their journal *Philosophical Transactions* in 1,665 [4]. The concept of pre-publication “peer review” dates to 1731, with the founding of *Medical Essays and Observations* [5]. The journal editor sent article copies to those deemed appropriately knowledgeable, albeit with the initial understanding that the process was designed only for article selection, rather than validation [5]. The Royal Society of London adapted this methodology in 1752, installing a pre-publication review committee that decided publication *via* a secret ballot. Throughout the next two centuries, political and economic change drove the exponential expansion of scientific research [6]. At the turn of the 19th century, the industrial revolution and technological advancements brought a concurrent and dramatic increase in research output [6]. In 1886 Thomas Henry Huxley described scientific periodicals as “a record of the progress of the sciences,” by which point there were 1,400 distinct journals [7]. In 1893, the editor-in-chief of the *British Medical Journal* was the first to utilise external reviewers with relevant knowledge for the qualitative analysis of manuscripts [8]. It was at this point that the contemporary concept of peer review was developed from 18th century roots, with the aim of elevating papers to the highest possible standard, reducing errors and improving content through a constructive process [5, 9]. Whilst economic crises in the USA and Europe around this time caused a relative depression in the growth of research, the end of the second world war brought about a new era of expansion [6]. At this stage, most reputable journals had adopted peer review [5, 9]. Remarkably, as of 2024, *Philosophical Transactions* is still running; its longevity underlines the fundamental importance of scientific journals in recording and sharing scientific history, current research, and future work.

### The Expanding Research Paper Landscape

It is thought that since the 1950s the growth in scientific papers has been exponential [6]. Due in part to this rapid growth, it is difficult to determine the exact number of research journals. “Scientific journal” is a loosely defined term, with new journals being created continuously [10], and others struggling and disappearing [11]. The advent of open access journals and the concurrent transition of many journals from print to exclusively online formats means that there is a wealth of online journals that can be accessed, greatly expanding global publication volumes [12]. In 2019 there were more than 20,000 open access science journals alone [12]. An analysis by Bornmann, Haunschild and Mutz estimates the exponential growth of publications at around five percent per year. The globally utilised biomedical literature database PubMed [13] deals with around one million papers annually, and estimates that the total number of published papers has been rising by between eight and nine percent per year over the past 30 years [14]. These differing figures highlight that this unprecedented growth proves difficult to track, despite the ease of data collection facilitated by the digital age.

### Challenges Facing Researchers and Peer Reviewers

Researchers are under increasing pressure to “publish or perish,” an expression that implies that the number and quality of publications is a key metric of research productivity [15]. This leads to a hyper-competitive research landscape, and publication volumes can be used as a key criterion when evaluating research grant applications [15, 16]. Publication volumes are also required for career progression, further incentivising the motivation to publish many papers. These pressures can lead to the submission of rushed, unnecessary or substandard papers for the sake of publishing, which exerts pressure on the peer review system. Reviewers are typically researchers themselves, peers with the expertise necessary to critically evaluate work within their field. As such, researchers spend increasing amounts of time working on primary research and writing papers, which means that less time can be devoted to peer review. This in turn increases the burden on other reviewers, leading to a cycle of increasing author pressure and reviewer fatigue.

It is important to note that the growth in publication rates differs by field and publication medium [17], as does the way that peer review is implemented. Peer review needs to be effective enough to cope with the most prolific fields and journals. The digital age has facilitated increasingly sophisticated forms of peer review. However, a lack of standardisation and qualitative analysis in an increasingly broad publication landscape means that it is difficult to conclude which of these are effective, fair, and practical for all parties involved.

## Current Peer Review Practice

### Aims of the Peer Review Process

The aim of the peer review process is to help journal editors assess which manuscripts to publish, excluding papers that are not on topic or that contain serious scientific flaws. In parallel, the peer review process must ensure that articles are legitimate (*i.e.*, not plagiarised or manufactured from fake data) [18]. Critically, it serves to elevate the quality of manuscripts to the highest standard that is practically possible [19]. The peer review process is intended to be constructive, optimising the chances of producing publishable material while also allowing authors to learn from the reviews [9, 19]. Rejection rates are high [20], but a constructive review process ideally facilitates improvements of rejected manuscripts that can then be submitted to other journals in a better shape. As well as the quality of research data interpretation, reviewers also assess the clarity of scientific writing. In essence, readers of scientific papers need to be reassured that an article that has passed peer review is an accurate addition to recorded science [21–23].

### The Typical Peer Review Process

The first stage of the process is manuscript submission ideally to a journal in an appropriate field. The manuscript then receives a preliminary, non-qualitative editorial review to ensure that it adheres to journal-specific formatting guidelines [18]. Manuscripts that pass this stage receive a second, qualitative review to ascertain whether or not their content is suitable for the journal. Subject to passing this stage, they will either be deemed appropriate for peer review by the journal editor, or if the paper is of insufficient quality, rejected outright; the latter is known as “desk rejection.”

The second stage is the selection of suitable reviewers. This is generally carried out by a member of the journal’s editorial board, sometimes aided by editorial assistants. Reviewers are generally chosen for their experience and knowledge in the relevant field, and because of their track record as reviewers. This is often determined through past interactions with the journal.

Some journals allow authors to suggest relevant reviewers, but there is the potential for bias; it is at the editor’s discretion whether to consider reviewers suggested by authors. Reviewer number per manuscript is variable, though two is the generally accepted minimum. Once invited by the editor, it is the reviewer’s responsibility to decide, based on the manuscript, whether they are an appropriate reviewer. This decision also takes into consideration potential conflicts of interest or gaps in knowledge. After declining, reviewers may be asked to suggest a more appropriate reviewer.

Once a reviewer has accepted the assignment, they receive the manuscript in confidence including access to other documents such as tables, figures, and supplementary information. Depending on the journal, the completion of a structured questionnaire may be required, where aspects of the manuscript are ranked qualitatively, or specific questions are answered. Ultimately, a common standard is the completion of a free text report, in which the reviewer writes up a categorical evaluation of the manuscript, generally listing several major and minor points. This format helps the editor make a final decision.

Reviewers may take different approaches, but what they ideally share is performing a thorough analysis of the manuscript. Reviewers check experimental design and the data presented, the novelty of the material and significance to the field. As well as underlining experimental errors and unclear data and suggesting additional controls and experiments, reviewers also pick up erroneous referencing, and even incorrect spelling, and grammar.

**FIGURE 1:**
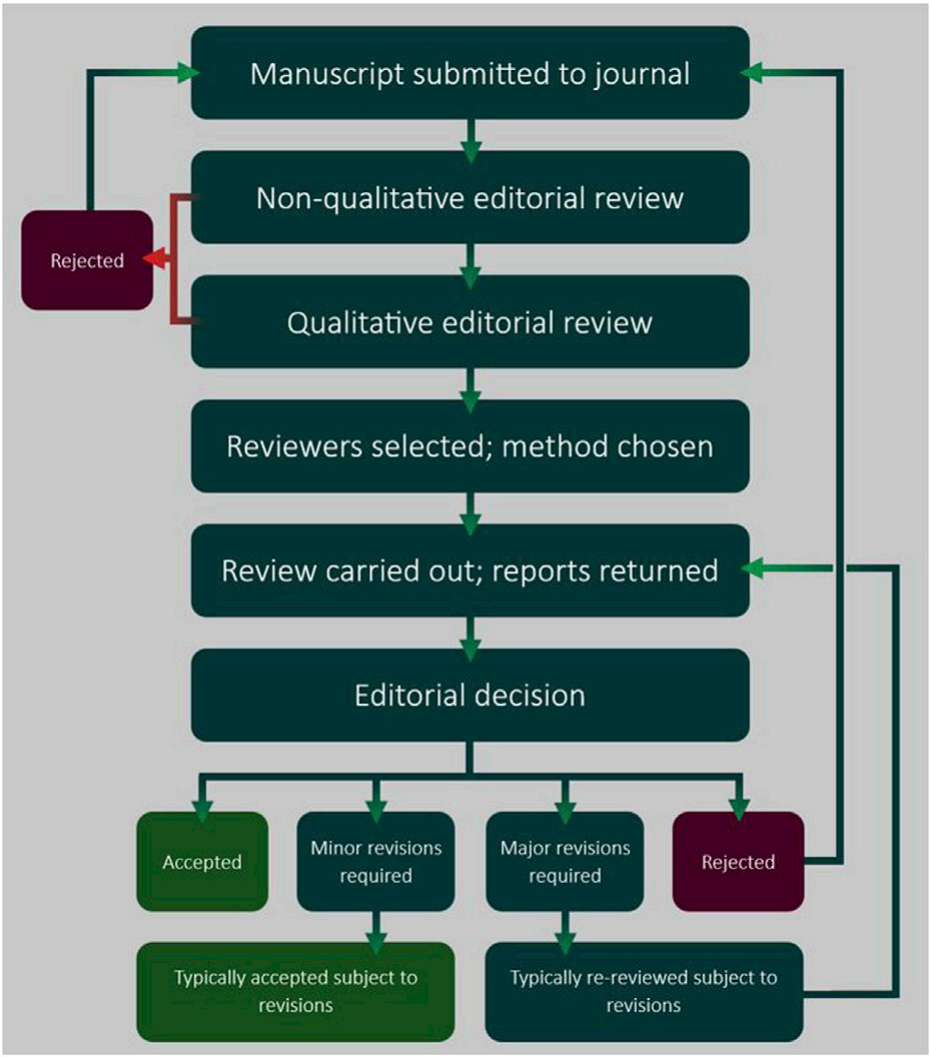
An overview of the peer review process. A typical peer review workflow is presented, illustrating the sequence of steps that are managed by the acting editor.

Following the return of all reviewer reports, an editorial decision is made. If the manuscript is not rejected (or, very rarely, accepted immediately), the editor conveys to the authors a decision that either minor or major revisions need to be made. Depending on the revisions suggested, the corresponding author submits a revised manuscript, generally within a specified timeframe accompanied by a point-by-point “rebuttal letter” in which the corresponding author addresses each of the reviewers’ comments. Typically, manuscripts with recommendations for minor revisions are accepted for publication subject to these revisions being met satisfactorily, whereas manuscripts marked for major revision often require another round of review. There does not appear to be a limit to number of additional rounds of revision, however policies may differ among journals. Authors may wish to withdraw the manuscript after multiple rounds if they feel that the process has become too lengthy or that the demands unable to be met [24]. Once the reviewers and editor are satisfied, the formatting and publication process is initiated. The typical peer review process is summarised in Figure 1.

### Different Peer Review Methodologies

There are numerous methodologies, both well established and novel. Single anonymous review is defined by anonymisation of reviewer identity, without anonymisation of author identity. Reviewer anonymisation ostensibly protects them from potentially unfair criticism, ensuring the rigour of reviewer comments [25]. Conversely, it is argued that this approach prevents accountability, and that the disclosure of author identity can lead to reviewer bias. As such, there appears to be a movement away from this approach in some journals. Despite these limitations, it is still amongst the most widely used method due to ease of implementation and fewer administrative steps involved.

Double anonymous peer review is defined by anonymisation of author details, and was previously known as double blind review. Neither party knows the identity of the other at any point during the process [26]. First introduced in 2001 by the journal *Behavioural Ecology*, this methodology is a relatively new addition to the research world [27]. In recent years more journals have adopted this method, and in some fields, it is the predominant peer review method [28, 29]. There is evidence that double anonymisation reduces targeted biases associated with identity, such as selection against authors of a particular gender or institution [26, 29]. As such, this methodology could preclude bias against specific groups of authors [29].

Triple anonymous review takes the extra step of anonymising the handling editor. This means that all three parties do not know each other’s identity. The editor holds significant power in regard to administrative decisions, so triple anonymisation aims to remove bias at editorial level. This methodology is new, and its implementation remains limited. Anonymisation needs to be maintained throughout the process, so there are extra administrative steps that may prove problematic, preventing its wider adoption.

Open and transparent review are often mentioned interchangeably, and as such, there is some confusion between the two. Here, open review is defined as any methodology in which both the author and reviewer are aware of the identity of the other, *i.e.*, there is no anonymisation process. This is sometimes referred to as “open identities” peer review, or “no-blinding” [30, 31]. In contrast to anonymous review, open designs are presumed to increase the accountability of reviewers. This may incentivise more careful and objective reviewing, resulting in fairer responses to all authors [32].

Transparent peer review features various aspects of transparency not seen in “closed” methodologies. This typically means that reviewer reports and author responses are published alongside the article [33]. As mentioned previously, transparent, and open review are often used interchangeably within the literature [34]. Reviewers who use the transparent approach are not obliged to disclose their identity to the authors, unlike mandated disclosure seen in open reviews [35]. Whilst reviewers are not obliged to disclose their identity, this approach may also incentivise more thoughtful review practice as the reports are public. The approach also allows the reader to assess whether the review process has been fair.

Collaborative review is a term encompassing models that involve crosstalk, either between reviewers, or between reviewers and authors throughout the review process [28]. This results in a review process that is potentially more constructive [28]. Like triple anonymous review, this methodology is very new and there are few examples of its implementation. Potential problems associated with this approach include issues around the time and effort required, and the ease and efficiency of communication between parties that are not, after all, established collaborators.

Post-publication review is a novel methodology whereby papers are evaluated and reported on after being formally published [36]. This can involve invited or voluntary reviewers, or third-party comments depending on where the paper is hosted; reviewers may be named or anonymous. This method has the advantage of quickly disseminating crucial research. An appropriate example regards the recent COVID-19 pandemic. Particularly in the earliest stages, it was imperative that research be shared and discussed rapidly to expedite the development of epidemic management, treatments, and vaccines. Whilst arguably helpful in distributing research rapidly, this is a novel method with little standardisation, and it could allow poor-quality research into the scientific record.

## Problems Associated With the Peer Review Process

### Challenges Associated With the Expansion of Research

Peer review is, as is evident, a long-standing and primary form of quality control of research outputs [9, 37]. Peer reviewed journals are the only periodicals to receive impact factors, and new scientific concepts and discoveries are not widely disseminated unless published in one. Nonetheless, there are key concerns about its overall efficacy and fairness. One concern is around the author/reviewer relationship and potential biases. Another is the lack of standardisation of practice, and the impact this has on its efficiency and objectivity.

The expanding research landscape is challenging for both researchers and reviewers. This is largely due to decreased time available to reviewers, whilst under pressure to work on their own publications. This has a cyclical impact, whereby pressured reviewers stop reviewing, and consequentially more pressure is placed on the remaining pool. There is a lack of reviewers to cover the literature [24] which has worsened since the *COVID-19* pandemic [38]. A professional, thorough, and objective evaluation process is expected, helping journals to process increasingly large volumes of submissions in good time, and allowing authors to learn from the process [18]. Whilst biases are impossible to remove entirely, it is important to find ways to minimise them. An effective system must balance efficiency and objectivity with fairness towards both authors and reviewers.

The peer review methodology that is chosen varies considerably across journals and in different scientific fields [28, 39]. An overview of Wiley Journals, for example, shows that double anonymous review comprises 65% of peer review models across all titles. At a field level, it ranges from 4% in physical sciences journals, to 26% in health sciences journals, with single anonymous comprising most of the remainder [28]. There is also variation within each review method, particularly with regards to more novel approaches. There is also a certain lack of a common understanding of definitions. A study by Ross-Hellauer, for example, found that there are twenty-two distinct definitions of “open peer review” in the literature [31]. A lack of consensus around definitions leads to difficulty in comparing methodologies.

### Objectivity and Bias

An effective system relies on efficient, unbiased analysis by the reviewer. Peer review is almost universally unpaid, so there is no financial incentive. Reviewers typically volunteer as it allows them to keep up with research in their field, and the task is an attractive addition to their CV. Many commit to reviewing properly and as fairly as possible as they feel duty-bound to aid other scientists, and crucially, expect reciprocation. Despite professional obligations and the collective need for a collegiate approach, there is still potential for bias and conflicts of interest. This is especially problematic in a changing and ultra-competitive “publish or perish” research climate. Reviewers in this system are under pressure from increasing workload and decreasing time to review, and are often *de facto* competitors, chosen for the relevance of their knowledge. Additionally, editors are part of the peer review process as adjudicators and can also be the source and subject of bias.

Various forms of bias exist that may impact both objectivity and efficiency. These are generally split into explicit “conscious” bias, and implicit, “unconscious” bias [40]. Bias in peer review is complex and difficult to quantify. Both forms of bias are damaging; implicit bias is likely much more common. Whilst reviewers and editors try to remain objective, unconscious bias can go unnoticed. Important sources of bias include gender, race, and affiliation (the prestige of the host institution, for example).

There is evidence of gender disparity within the research sphere. While the participation of women in STEM fields is increasing, publication output remains skewed in favour of men [41]. Most primary authors are male, and there is a concurrent lack of representation of women as reviewers and editors [41]. The reason for this discrepancy is complex in nature, but it is important to establish whether the peer review process is affected by gender bias. There is diminished representation of women in administrative roles such as editorial boards, and in peer review itself. This is not only important from a perspective of equality, but also from the standpoint of reviewer burden. Lack of women in these roles may be contributing to shortages, and thus increasing pressure on the system. Some surveys suggest that women avoid submitting manuscripts to certain journals because of a perceived bias favouring men [41]. It is important that the extent of gender bias and inequality in the peer review system is properly understood and tackled so that the peer review process can become more inclusive and effective.

Another important aspect of bias in peer review is author affiliation. Prestige bias relates to an author’s reputation, either with respect to individual notoriety in the field, or the author’s institution [42]. There is evidence that authors from higher income countries or more prestigious institutions receive more favourable review reports, relative to those affiliated with poorer countries or less renowned research institutes [43]. Furthermore, acceptance rates are demonstrably and significantly higher when the author’s name and affiliation are not anonymised [44]. There is positive bias for authors and institutions within the same region as the reviewer, along with the assumption that wealthier institutions and countries produce more reliable research. There may also be bias against writing styles that are the expression of a non-English native language speaker. Ideally, proper peer review focuses on scientific matters rather than over-emphasizing imperfect writing (that can be improved at revision stage), the prestige of the author or institution is not a consideration. Where there is bias, papers may be rejected that could otherwise be improved to a publishable standard. An interesting consideration is that 10 percent of reviewers are responsible for 50 percent of reviews, and that most peer reviews are written by researchers from developed countries [45]. The latter is, no doubt, partly explained by the fact that there is more research in developed countries. Might there be some bias in some circumstances against authors and peer reviewers from developing countries?

Additionally, there are contentious issues around the income sources of journals, and the way that research is monetised. Geographically speaking, the publishing sector is predominately led by a small number of North American and Western European corporations; these are said to impart pressure on government bodies to restrict copyright laws and increase the monetisation of intellectual property signed over to them by authors [46]. In this scenario, institutions take the hit for these fees, usually via a subscription model. There has been an increasing drive to switch to Open Access (OA) publication models. OA publication is defined by online repositories of published research that are freely accessible, without access charges [47]. This helps the accessibility of research; however, it does generate a contentious issue. OA publication is generally associated with article processing charges; these can become unaffordable for individual researchers. The fact that these are charged at an article level rather than at a journal level allows journals to take advantage of the system for monetary gain. This arguably encourages the proliferation of lower quality journals built for profit. These include unethical “predatory” publishers that unreasonably profit from authors financially [48]. Such predatory publishers are often hard to spot, and care needs to be taken by authors to avoid them [49]. The OA system also arguably favours wealthier, developing countries where authors are more likely to have the money to pay for these charges.

Editors are also part of the system and are also potentially affected by bias. The editor is responsible for the selection and invitation of reviewers. Ideally, editors choose reliable, objective reviewers with no conflicts of interest; however, they may not always properly implement a fair reviewer selection process. Authors may suggest their own reviewers, but there is the possibility that reviewers suggested by authors are more likely to be biased [50]. Some editors may even suggest purposely opposed reviewers to attempt a balanced review, though this also risks bias as it could favour overly critical reviews [19]. The editor is responsible for making a final decision based on the reviews and communicating it to the authors. Some reviewers may suggest unreasonable, excessively time consuming and even conflicting changes. Unnecessary and excessive comments could waste an author’s time, especially if they involve lengthy additional experiments that do not significantly enhance the quality of the science or are beyond the realistic scope of the manuscript. Reviewers are also at times in disagreement, and in such cases, it is the editor’s responsibility to make a fair decision—this is not always straightforward. Editors are also responsible for pre-review article appraisal and know the author’s name; this could already cause bias for or against authors. Editorial bias can also affect post-review decisions [51]. Editors can seek extra reviewers or consult other editorial staff prior to deciding, but they may not always exercise these powers and instead favour their own opinion [19]. Editors may not always have the time or inclination to scrutinise the fairness of each review. Hence biased reviews may filter through, because editors are not scrutinising reviews sufficiently, egged on by journals demanding quick processing times.

Another contentious question is the potential theft of intellectual property. Peer review entrusts reviewers with confidential information. Whilst rare, “predatory reviews,” whereby reviewers suggest rejection and steal ideas for their own gain, are difficult to spot. There was even a case in which an entire paper was stolen by a reviewer [52]. These incidents can be extremely damaging to the authors and to the integrity of the process [53].

It is vital that reviews are performed objectively, providing a fair judgement of manuscripts without bias for or against the author. Negative bias clearly has an impact on inclusivity and can skew the published literature. Positive bias may lead to publication of poor-quality papers that have not been objectively moderated. Both types of bias can negatively affect the peer review process.

### The Efficiency of the Peer Review Process

Whilst a single review takes on average only 6 hours [9], manuscripts can remain on desks for long periods before editorial or reviewer evaluation. One-third of papers are left for 2 weeks, and one-sixth for a month or more prior to desk rejection [24]. Given the number of stages involved any delay is cumulative; and the entire process can become unnecessarily lengthy. Total review time averages range from 12–14 weeks in medical, public health and natural science journals, to 25 weeks or more in other fields, such as economics and business journals [24]. In extreme cases it can even take over a year to evaluate and publish a manuscript [54].

Delays in the peer review process have only worsened since the *COVID-19* pandemic, further stressing the system. Furthermore, higher impact factor appears to correlate with lower first response time. This could be due to differences in organisation and resources as well as, presumably, variation in how thoroughly journals assess manuscripts [24]. Impact factor serves as a metric of journal quality, and high impact factor journals typically handle groundbreaking research. Overall, these differences highlight the fact that there is a great deal of variability in how efficiently the process is carried out. Delays can be down to administrative factors, where the method chosen impacts the length of the peer review, and delays are also a result of the hectic professional lives of reviewers. The editor has a responsibility to keep the process running smoothly; however, editors often struggle to find reviewers, and unreasonable time constraints set by journals can prevent reviews from accepting tasks or even cause them to quit the review process. Delays can impact authors greatly, preventing the timely publication of important results, and can even allow competing researchers to publish first [53, 55]. The benefit of improving efficiency is two-fold; researchers can publish research in a more expedient manner, while also giving them more time to review other work.

Furthermore, journal standards differ. Reviewers, therefore, often evaluate relative to the journal’s standards and expectations. This has been described as “quality censoring;” in other words, the effort expended by the reviewer is arguably proportionate to the journal’s standard, rather than the reviewer’s perspective [56]. There is also no systematic, formal training for reviewers to ensure that they are objective and efficient [57]. It is difficult to evaluate the standards of objectivity when there is no formal training, and when there are thousands of journals with different approaches to the peer review process.

### Efficacy and Reliability of the Peer Review Process

Finally, a fundamental question relates to the overall efficacy of the peer review process. It is difficult to quantify efficacy objectively, especially given the number of factors and stages involved in the process [9]. Alongside objectivity and efficiency, the accuracy of the peer review process is questionable, especially in a system where reviewers and authors are under pressure. Studies in which intentional flaws were introduced into papers found that most errors are missed. In a striking 1998 study, Godlee, Gale and Martyn deliberately added eight errors to a paper, finding that none of the established reviewers used spotted more than five of the eight [58]. Furthermore, poor quality research is still published despite peer review. Some enterprising IT students created a program, SCIgen, to generate “nonsense” papers; following submission to conferences these ended up in peer reviewed journals [59]. SCIgen became open source, and some authors even started to use it to boost publishing output artificially. The ability of these fake papers to get through peer review is very worrying [59].

There is of course the process of retraction, whereby papers are pulled post-publication; this can happen because of honest errors or intentional research misconduct. Retraction is imperative in maintaining literature standards and incentivising good practice; however, it is not infallible. The process can also be damaging to authors. COPE, the Committee of Publication Ethics, have established themselves as an authority on good retraction practice, publishing a list of guidelines that their thousands of member journals should try to adhere to [60]. One COPE guideline suggests that a clear justification should be given for a retraction, particularly to differentiate retractions due to honest errors from those linked to research misconduct. In practice, journals do not always provide such statements, with around 10% omitting information on why the retraction was initiated [61]. Consequently, papers retracted due to honest errors may be assumed by peers to have been retracted because of poor scholarship or even misconduct, harming author reputations. Finally, retraction does not always fully remove flawed research. A 2020 study by Schneider *et al.* scrutinised a paper published in 2005 and retracted in 2008, finding that the paper was continuously cited throughout the period studied, 2006–2019. 96% of these citations failed to mention the retraction [62], which suggests that retraction notices do not prevent the dissemination of erroneous data. One study of BioMed Central journals during the period 2000–2015 found that the median time between publication and the 134 retractions considered was 337.5 days [63]; in other words, papers with clear errors were accessible for almost a year before retraction. Overall, the retraction process provides a helpful safety net for the removal of clearly flawed papers; however, its shortfalls highlight the necessity for strong, pre-publication processes.

### The Impact of Differing Methodologies

The double anonymous peer review processes have the advantage of reducing the potential for explicit biases, such as institutional and gender bias. Conversely, it could be argued that author identification is important for verifying author reliability. Additionally, author identity can allow a reviewer to contrast the manuscript with their previous work, validating any claims or points of contention [9, 18]. Whilst double blind review may provide better protection against bias, there is mixed evidence as to how authors react to it. Some surveys suggest that authors prefer double blind review [64], and it has become the predominant review methodology in some fields [28]; however, its adoption remains low in other fields [65]. Importantly, double anonymous review carries extra administrative burdens, which may be unfavourable in a pressured system. The latter may explain why single blind review remains predominantly in use, despite the increased risk of bias against authors.

Triple anonymous review takes the step of anonymising the editor to all involved parties, as well as the identity of the author and reviewer to the editor. This precludes identity-based biases, such as prestige, institution, and gender bias. This however carries even more administrative work than other anonymous review methods, and therefore its adoption is infrequent. Given its infrequent use, it is hard to assess its impact on objectivity and efficiency.

Open review design is potentially advantageous as the reviewer is held more accountable for unfair or unrealistic comments, and for an unreasonable delay of the process. On the other hand, open reviews may incentivise censored or biased analysis. Some reviewers may withhold valid criticisms to avoid conflict or judgement. Reviewers that favour the author may leave unduly positive reviews and similarly, reviewers that do not like the author may produce unduly negative reports. Overall, an open reviewer-author dynamic may bring about prestige biases. This approach may also put additional pressures on reviewers; increased reviewer accountability may serve as a deterrent for reviewers, further straining a limited reviewer pool.

## Future Directions

Whilst several problems associated with the peer review process are evident, it is undoubtedly the case that it remains the best way to moderate scientific literature: it enhances quality and removes unpublishable research. Several improvements to the process have been suggested, as follows.

### Open Peer Review Approaches

Open peer review is an interesting concept. Open reviewing could make reviewers more accountable; however, it could also encourage timid reviews. This could be especially likely when reviewing work from a highly respected expert in the field. Two forms of open review being trialled are post-publication and dynamic review. Open forms of post-publication review allow continuous discourse on published papers, whilst still holding reviewers accountable [66]. Journals may invite trusted reviewers or moderate reviews by only allowing reviewers with a minimum number of their own publications; however, this could be limiting to new reviewers. Whilst this methodology is promising, there are limitations. This is a novel method with low participation rates, meaning that not every reviewable paper receives post-publication comments. Furthermore, automated reviewer moderation may not be sufficiently thorough, as there are no checks for relevance of reviewer expertise. Reviewer commentary can also be held on multiple sites depending on which hosts facilitate post-publication review; this can cause the discussion to be inconsistent and fragmented [36]. Dynamic open review operates similarly but utilises online “preprint” repositories to allow reviews on pre-publication manuscripts. These are dynamic online archives to which updated manuscripts can be added by authors, making large volumes of research available for review, and where reviewer comments and author edits can be added in real-time. Both post-publication and dynamic review also help prevent intellectual property theft as reviews are conducted on manuscripts that are already accessible.

Another alternative process is the “transparent” review. This aims to publish the entire process, including editorial, reviewer and author correspondence; some journals define this simply as the concomitant publication of anonymous reviewer reports alongside manuscripts [33]. The journal *Genome Biology* has implemented transparent review permanently. The journal maintains that concerns such as the reluctance of reviewers are unfounded, and that their readership is provided with reports to evaluate reviews [33]. These transparent review processes could potentially prove effective; however, it is currently difficult to compare them empirically to the established peer review practices, given low current use of the method. More fundamentally, the principle of anonymity is widely seen as an essential part of the review process, and for good reason: it is shown to be essential in preventing bias, retaliation, and other unethical practices.

### Reviewer Training and Accreditation

Another approach to improving the peer review process is to focus on the quality of the reviews. One suggestion is to provide formal training of reviewers. A survey conducted by *Sense about Science* suggested that around 56% of reviewers feel that there is insufficient guidance on how to review, and that 68% believe training would improve review quality [57]. Despite this, some attempts at training did not make a significant difference, perhaps because participants were long-time reviewers with ingrained habits [54]. Early career reviewer training may be more beneficial. Most reviewers learn as young scientists by observing mentors [9]; this could be an appropriate time to offer formal training in peer reviewing. Ultimately, the early teaching of peer review skills cab help to increase the pool of available reviewers and promote good practice early in a career. There are, of course, several practical problems—who will offer the training? And would there be a universally accepted set of training criteria?

Another suggestion is to offer reviewer accreditation, reward, or acknowledgement to incentivise higher standard reviews, and to encourage more reviewers to take up reviewing. Some journals offer accreditation via a points-based system [66]. However, such approaches could incentivise excessive reviewing, for example, of papers that are not suitably matched to expertise. To counter this, some journals base accreditation on variables such as number of outstanding reviews per year, as judged by editors [67]. This may incentivise higher standards of review; though ultimately this is subjective, and prone to quality censoring by editor or journal standards. This also increases administrative load on journal editors. Some journals accredit via profiling platforms such as Publons, where open records of reviewer contributions are kept for sharing with others or to attach to a CV [68]. The integration of these open, accessible systems with an open review may prove to be an interesting approach to test new peer review models. There is also a suggestion that financial rewards could expedite review times and increase participation. The counter argument is that a financial reward could decrease review reliability by shifting reviewers’ motivations from being collegiate and altruistic to being selfish [69]; therefore, non-financial incentives may prove to be more effective than financial rewards.

### The Collaborative Review Approach

Finally, the collaborative review approach combines the desire to improve review methodology and reviewer efficacy. The basic concept is to utilise online real-time editing and commentary tools to allow multiple reviewers to evaluate a manuscript in a conversational manner [70]. In a trial by *Molecular Cell*, the majority of the 17 of 24 reviewers who responded to a survey showed willingness to take part in such discussions; and of 10 authors, five responded positively [70]. Some journals are trialling a mixed collaborative process, incorporating aspects of the open review concept [71]. This approach potentially provides a structured, interactive, more informal, and faster process in which all parties can converse with each other transparently [71]. Whilst this may risk the unwillingness of participants to contribute fully because they wish to avoid conflict, moving to a more informal, open, and conversational system may prove more constructive than closed systems. The collaborative review approach could also incorporate reviewer accreditation through a formal acknowledgment in the ensuing publication. As discussed by Hoffman, key aspects highlighted by this review methodology are trust and accountability. An open discussion between author and reviewer could reduce the chance of unfair or biased comments, thanks to increased accountability from direct communication with the author. An informal, two-sided approach could also build trust between groups of reviewers and authors and provide greater depth of discussion which may potentiate more open, honest research. This approach may also increase review efficiency, by avoiding issues inherent in traditional processes, such as lengthy, repeated rounds of review and prolonged response times. Despite this potential, the implementation of the collaborative review is limited, and so it is difficult to evaluate how effective it may be relative to traditional review methodologies.

### The Use of Artificial Intelligence

Whilst technological advances have facilitated an increase in publication output and thus put more strain on the system, a promising advancement in the technological world is the growing use of Artificial Intelligence (AI) techniques. Estimates suggest that as of 2018, 15 million hours are spent each year on review of previously rejected manuscripts [72], and this figure is likely rising as the research world continues to expand. It is suggested that AI could be used for administrative tasks; indeed, AI techniques are already in use for some administrative checks such as plagiarism screening and manuscript compliance [72]. Cutting out the wasteful review of manuscripts that may be more suited as submissions to other journals can save a significant amount of time and effort on behalf of the reviewer, and will allow more time to review more appropriate submissions. Additionally, it is suggested that AI might be useful in matching reviewer to manuscript and manuscript to journal regarding relevance of knowledge, streamlining an inefficient process and taking administrative load away from editorial staff [72]. However, clearly AI models must be trustworthy, and there must be confidence that the algorithms used do not bring about new biases. Reliance on AI especially without prior testing may create new problems, and its use therefore needs to be evaluated very carefully.

## Conclusion

The peer review process remains an integral part of the publication of scientific papers, but its implementation is sometimes problematic, and it is therefore appropriate to explore how it could be improved. Challenges include the lack of availability of reviewers because of the sheer volume of papers being processed nowadays. Another significant problem is bias, both conscious and unconscious, against individuals or institutions. Several novel peer review approaches have been proposed and piloted; and together with advances in technology such as artificial intelligence, there are many opportunities to improve established peer review practice. Reviewer training, perhaps early in a career, could also improve peer review, however there are understandable challenges regarding its implementation. The main challenges are varied implementation of new ideas around peer review, lack of widespread adoption of novel methodologies, and therefore limited understanding of the efficacy of new approaches. Despite these caveats, there is increasing awareness that the peer review process can and ought to be improved, ultimately leading to a better, fairer, and more effective process.
